# Azithromycin Exhibits Activity Against *Pseudomonas aeruginosa* in Chronic Rat Lung Infection Model

**DOI:** 10.3389/fmicb.2021.603151

**Published:** 2021-04-23

**Authors:** Manoj Kumar, Madhvi Rao, Tarun Mathur, Tarani Kanta Barman, Vattan Joshi, Tridib Chaira, Smita Singhal, Manisha Pandya, Souhaila Al Khodor, Dilip J. Upadhyay, Nobuhisa Masuda

**Affiliations:** ^1^Department of Microbiology, Daiichi Sankyo India Pharma Private Limited, Gurgaon, India; ^2^Research Department, Sidra Medicine, Doha, Qatar; ^3^Department of Pharmacokinetics and Metabolism, Daiichi Sankyo India Pharma Private Limited, Gurgaon, India

**Keywords:** Gram-negative bacteria, PA-14, PAO1, multidrug resistance, respiratory tract infection, *pel* genes, extracellular biofilm matrix, quorum sensing molecule

## Abstract

*Pseudomonas aeruginosa* forms biofilms in the lungs of chronically infected cystic fibrosis patients, which are tolerant to both the treatment of antibiotics and the host immune system. Normally, antibiotics are less effective against bacteria growing in biofilms; azithromycin has shown a potent efficacy in cystic fibrosis patients chronically infected with *P. aeruginosa* and improved their lung function. The present study was conducted to evaluate the effect of azithromycin on *P. aeruginosa* biofilm. We show that azithromycin exhibited a potent activity against *P. aeruginosa* biofilm, and microscopic observation revealed that azithromycin substantially inhibited the formation of solid surface biofilms. Interestingly, we observed that azithromycin restricted *P. aeruginosa* biofilm formation by inhibiting the expression of *pel* genes, which has been previously shown to play an essential role in bacterial attachment to solid-surface biofilm. In a rat model of chronic *P. aeruginosa* lung infection, we show that azithromycin treatment resulted in the suppression of quorum sensing-regulated virulence factors, significantly improving the clearance of *P. aeruginosa* biofilms compared to that in the placebo control. We conclude that azithromycin attenuates *P. aeruginosa* biofilm formation, impairs its ability to produce extracellular biofilm matrix, and increases its sensitivity to the immune system, which may explain the clinical efficacy of azithromycin in cystic fibrosis patients.

## Introduction

*Pseudomonas aeruginosa* is the most common bacterial pathogen that causes biofilm-mediated chronic lung infections among cystic fibrosis (CF) patients (Faure et al., 2018; Maurice et al., 2018). *P. aeruginosa* has an innate propensity to attach to different solid surfaces and form biofilms, which enables the bacteria to resist both the host’s innate immune system and treatments with antibiotics (Mulcahy et al., 2014). The treatment options for nosocomial Gram-negative infections are very limited. The antibiotics of choice for Gram-negative pathogens are parenteral carbapenems, such asimipenem and meropenemor quinolones (Bassetti et al., 2018). However, the poor activities of these antibiotics on bacterial biofilms and the increasing prevalence of multidrug-resistant *P. aeruginosa* (Cabot et al., 2012; Ciofu and Tolker-Nielsen, 2019; Kumar et al., 2019) leave the physicians with very limited choices to effectively treat these patients.

Although macrolides are commonly used for Gram-positive pathogens, azithromycin is being extensively used to treat chronic respiratory tract infections caused by *P. aeruginosa* especially in cystic fibrosis patients (Hansen et al., 2005; Wagner et al., 2005). In addition to the direct anti-pseudomonas effects (Tateda et al., 1996; Marvig et al., 2012), multiple clinical studies have highlighted the beneficial non-antibiotic effects of azithromycin, including modulation of the aberrant immune response and improving lung function in CF patients (Equi et al., 2002; Saiman et al., 2003; Clement et al., 2006; Principi et al., 2015; Cogen et al., 2018). Taken together, multiple mechanisms of azithromycin have been suggested, such as inhibiting biofilm formation by modulating the synthesis of quorum sensing (QS) molecules (Nalca et al., 2006; Swatton et al., 2016) or stimulating the anti-inflammatory effect during prolonged exposure (Zimmermann et al., 2018).

*P. aeruginosa* poses different QS signaling systems (LasI/LasR and Rh1I/Th1R)that regulate different cellular processes, including cell-to-cell communication, extracellular matrix synthesis, and biofilm formation (de Kievit and Iglewski, 2000; Smith and Iglewski, 2003). A large number of genes, including those involved in virulence and biofilm formation, are modulated by two acyl-homoserine lactone molecules synthesized by QS systems, namely, 3-O-C12homoserine lactone (HSL) synthesized by *lasI* and C4-HSL synthesized by *Rh1I* (Ding et al., 2018). The QS signaling molecule 3-O-C12 HSL has been shown to be essential for extracellular matrix modulation and biofilm formation since the *lasI* mutant was found to be defective in extracellular biofilm matrix and biofilm formation (Sakuragi and Kolter, 2007). In contrast, the Rh1I/Rh1R signaling system was found to be important for the survival of bacterial cells during anaerobic conditions in biofilms (Yoon et al., 2002; Sakuragi and Kolter, 2007). Interestingly, an increasing level of 3-O-C12 HSL has been reported in both *P. aeruginosa* biofilm formed *in vitro* and in CF patients chronically infected with *P. aeruginosa* (Geisenberger et al., 2000; Singh et al., 2000; Barr et al., 2015). Azithromycin has been shown to inhibit the synthesis of 3-O-C12 HSL signaling QS molecules (Favre-Bonte et al., 2003) without influencing the growth of planktonic cells (Chalmers, 2017).

However, data concerning the mechanism of biofilm inhibition by azithromycin and *in vivo* efficacy in chronic pulmonary infection, to support the clinical utilities of azithromycin in CF patients, are scarce. In this study, we evaluated the efficacy of azithromycin against *in vitro* and *in vivo P. aeruginosa* biofilm formation in a chronic pulmonary rat infection model at a human-equivalent dose. Our study revealed the potential mechanism of biofilm inhibition by azithromycin.

## Materials and Methods

### Bacterial Strains and Antibiotics

*P. aeruginosa* strain PAO1, PA-14 (highly virulent clinical isolate), and ATCC 700829 (standard strain for biofilm formation) were used for all *in vitro* and *in vivo* biofilm experiments. A recombinant *P. aeruginosa* G2 strain having a single chromosomal copy of promoterless *lacZ* gene, under promoter control of the *pelA* gene, was used for the mechanism of action studies. The bacterial isolates were obtained from the American Type Culture Collection (Manassas, VA, United States) or from our proprietary collection of clinical isolates. Levofloxacin was synthesized at Daiichi Sankyo Co., Tokyo. Azithromycin and clindamycin were obtained from a commercial source. All strains were stored at −80°C in 20% glycerol in trypticase soy broth (TSB) (Becton, Dickinson, and Company, Cockeysville, MD). All bacteriological media were procured from Becton Dickinson and Company; the reagents and chemicals were purchased from Sigma-Aldrich Co., LLC, United States.

### *In vitro* Biofilm Activity

The effect of azithromycin and clindamycin on *P. aeruginosa* biofilm formation was assessed as described previously (Sharma et al., 2009; Barman et al., 2016; Kumar et al., 2019). Briefly, the overnight-grown culture of *P. aeruginosa* PAO1 in TSB was pelleted and resuspended in Luria–Bertani broth containing 0.2% glucose (10^7^ CFU/ml). The bacterial suspension was spiked with different concentrations (16–0.015 μg/ml) of azithromycin or clindamycin in 96-well plates and incubated at 26 ± 2°C. The effects of azithromycin and clindamycin on inhibition of biofilm formation were assayed after 24 h of exposure. The wells were emptied and washed three times with phosphate-buffered saline (PBS; pH 7.3), and the adherent biofilm was stained with 200 μl of 1% crystal violet for 30 min and rinsed with distilled water. The adherent biofilm in the wells was solubilized with 200 μl of 30% acetic acid, and the optical density at 600 nm (OD_600_) was measured to quantify the formed biofilms. Each experiment was performed thrice, and the mean percent inhibition and standard deviations were calculated. The relative inhibition of biofilms, expressed as a mean percentage, was determined using the following formula:

$$\mathrm{Percent}\,\mathrm{inhibition}=100-\left\{\left(\frac{O\,D_{600}\,o\,f\,d\,r\,u\,g\,w\,e\,l\,l}{O\,D_{600}\,o\,f\,p\,o\,s\,i\,t\,i\,v\,e}-\text{control}\,\text{well}\right)\times 100\right\}$$

The efficacy of azithromycin was also evaluated on a pre-formed biofilm of *P. aeruginosa* PAO1 to assess the therapeutic or eradicative effect of azithromycin as described earlier (Macia et al., 2014). The bacterial suspension was prepared as described above and inoculated at 200 μl/well in a 96-well plate. After 24 h of incubation, the wells were emptied and washed with PBS and then spiked with different concentrations (16–0.015 μg/ml) of azithromycin prepared in LB broth containing 0.2% glucose. The plate was further incubated for 24 h, and the effect of azithromycin was assessed as described above.

To further evaluate the effect of azithromycin on planktonic cells and biofilm formation, a time-kill kinetic study was performed. Briefly, 10 ml of LB broth containing 0.2% glucose and a sterile 2-cm-long piece of the catheter were pre-warmed at 26 ± 2°C in reaction tubes, and the different concentrations of azithromycin (2 or 16 μg/ml) were added to tubes. The bacterial inoculum of *P. aeruginosa* PA-14 was prepared as described above and added to the tubes to achieve a final inoculum of ∼1 × 10^5^ CFU/ml. The tubes were incubated at 26 ± 2°C. The planktonic cell counts were determined at various time-points by serial dilution plating onto a trypticase soy agar plate. After 24 h of incubation, the catheter pieces from each tube were removed, non-adherent bacteria were washed from the catheter pieces, and the number of CFU embedded in biofilm per catheter was determined as described earlier (Kumar et al., 2016, 2019). The experiment was performed in triplicate, and the mean log_10_ killing of planktonic cells and biofilm inhibition were calculated for each concentration. To further assess biofilm formation in the absence (drug-free control) or presence of azithromycin, the catheter pieces were also sectioned and processed for scanning electron microscopy (SEM).

### β-Galactosidase Activity

To understand the molecular mechanism of biofilm inhibition, a recombinant mutant *P. aeruginosa* G2-containing promoterless *lacZ* gene under promoter control of *pelA* was gifted to us by Prof. Kilter (Sakuragi and Kolter, 2007). The β-galactosidase activity was performed in the absence and presence of different concentrations of azithromycin and levofloxacin, as described earlier (Sakuragi and Kolter, 2007).

### *In vivo* Studies

Specific pathogen-free female Sprague–Dawley (SD) rats, weighing 200 ± 20 g (age 8 weeks), were used for the study. The rats were procured from Vivo Biotech Pvt. Ltd., Hyderabad, India. The animals were housed in a pathogen-free animal facility and allowed 5 days of acclimatization in the study room before initiating the experiment. Feed and water were provided *ad libitum* during acclimatization and the study periods. All animal studies were approved by the institutional animal ethics committee. The animal studies were conducted under the strict guidelines of the committee for the Purpose of Control and Supervision of Experiments on Animals, Ministry of Environment, Forests and Climate Change, New Delhi, Government of India, and under the supervision of the institutional committee.

To determine the *in vivo* efficacy of azithromycin, a chronic biofilm model *of P. aeruginosa* was established in SD rats, which mimic the human chronic infection (Hoover et al., 2017; Chawanpaiboon et al., 2019). A semisolid 0.25% w/v agar beads suspension was prepared and autoclaved a day before the *in vivo* experiment and stored overnight at 4°C. To cause a chronic biofilm infection, *P. aeruginosa* PA-14 was grown overnight on Mueller–Hinton agar (MHA; Becton, Dickinson and Company), and three to four isolated bacterial colonies were picked and suspended in PBS buffer to prepare the bacterial suspension. The turbidity of bacterial suspension was adjusted to 1 McFarland, diluted 1:10 with PBS. To prepare the inoculum, bacterial suspension was embedded in agar beads as detailed in a previously described method, which yield beads of mean size approximately 200–300 μm and a bead suspension of 2.5 × 10^7^ CFU/ml (Hraiech et al., 2012). The animals were anesthetized by injecting 100 μl of xylazine (5 mg/kg) and ketamine (100 mg/kg) mixture intramuscularly. Using an 18-gauge blunt needle, 150 μl of bead suspension was injected intratracheally as described earlier (Barman et al., 2011). To cause a chronic biofilm infection, the animals were kept untreated for 3 days. Before starting the treatment, the animals were randomized using their body weight, and *n* = 6 animals were kept in each group. Azithromycin was administered orally using agavage at 75 mg/kg, q24h for 4 days. The rats were then euthanized 22 h after the last dose, and the lungs’ bacterial load was determined by plating diluted lung homogenates on MHA.

### Pharmacokinetics Studies of Azithromycin in *P. aeruginosa*-Infected Rats

The concentrations of azithromycin were assessed in the plasma and lung epithelial lining fluid (ELF) of the rats infected with *P. aeruginosa* PA-14. Intratracheal infection in rats was performed as described above. A single dose of 75 mg/kg of azithromycin was administered orally through gavage. Blood and broncho-alveolar lavage (BAL) fluid (with 2 ml of normal saline wash) were collected at different time-points up to 24 h post-dose from each animal by gross dissection. For the collection of BAL, 2 ml of sterile cold PBS was infused into the animal through the trachea and into the bronchial cavity and aspirated out. Plasma was harvested from blood samples by centrifugation. The azithromycin concentrations in plasma and BAL samples were determined using liquid chromatography with tandem mass spectrometry. The apparent volume of ELF (*V*_ELF_) in BAL fluid was determined by the urea dilution method using the urea assay kit from BioChain (Hayward, CA). The concentration of azithromycin in ELF (*C*_ELF_) was determined as

$$C_{\text{ELF}}=C_{\text{BAL}}\times \frac{V_{\text{BAL}}}{V_{\text{ELF}}}$$

where *C*_BAL_ is the measured concentration of azithromycin in BAL fluid.

### Statistical Analysis

All data were analyzed using GraphPad Prism (version 8; GraphPad Software, San Diego, CA, United States). The statistical significance of the difference between the numbers of viable organisms recovered from the lungs of the treated group and those for the untreated control group or biofilm inhibition as compared to control (no drug control) was evaluated by non-parametric Mann–Whitney analysis. A difference between the treated group and the untreated control group was considered to be statistically significant if the *P* < 0.05.

## Results

### *In vitro* Biofilm Activity

**FIGURE 1:**
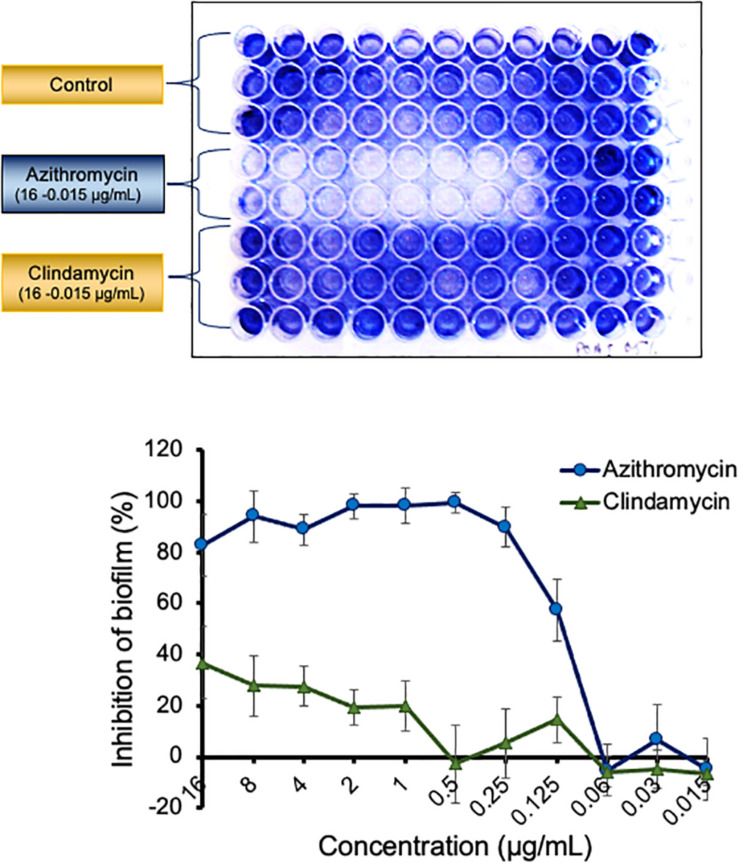
Activity of azithromycin and clarithromycin against biofilm of *P. aeruginosa* PAO1 The adherent biofilm in presence of azithromycin, clindamycin or in control well (drug free) was stained with 1% crystal violet and measured by OD_600_ after dissolving in 30% acetic acid. The inhibition of biofilm in presence of antibiotics was calculated by comparing the biofilm formation in control well. Values shown in the figure are average of three independent experiments.

Azithromycin exhibited a potent activity against biofilms produced by different isolates of *P. aeruginosa*. Azithromycin showed a strong biofilm inhibition as compared to the drug-free control and clindamycin with a biofilm preventive concentration (BPC_50_) value of 0.122 μg/ml against *P. aeruginosa* PAO1 (Figure 1) and also exhibited a similar biofilm activity against *P. aeruginosa* ATCC 700829 (standard strain used for biofilm formation) (Supplementary Figure 1). In contrast, azithromycin exhibited a comparatively reduced activity against the pre-formed biofilm with a minimum biofilm eradication concentration (MBEC_50_) value of 7.49 μg/ml against *P. aeruginosa* PAO1 (Supplementary Figure 2).

**FIGURE 2:**
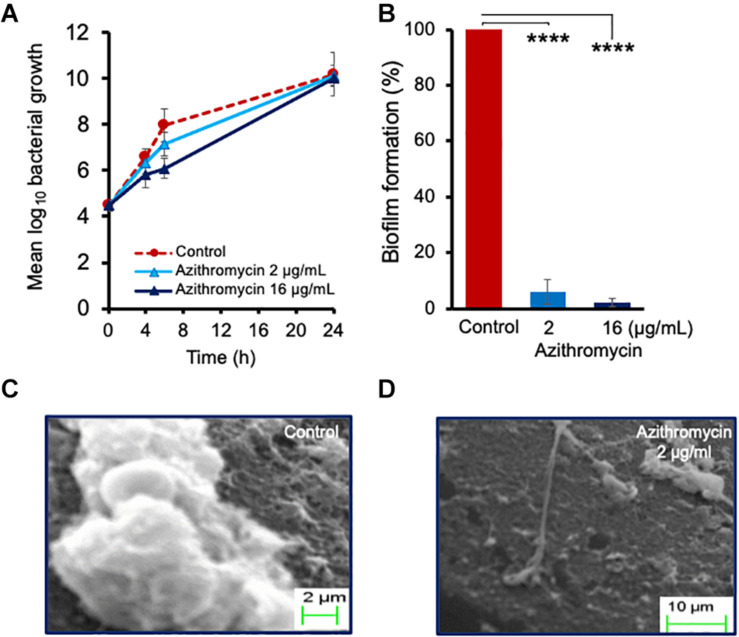
Activity of azithromycin against planktonic cells of *P. aeruginosa* PA14 **(A)**, and biofilm formation **(B)** at 24 h. Scanning electron micrographs revealing biofilm formation at 24 h in absence of drug **(C)** or in presence of azithromycin **(D)**. The catheter piece in drug-free control well shows production of extracellular matrix and biofilm formation, while no extracellular biofilm matrix and biofilm formation observed in presence of azithromycin. Each value is the average of triplicate assay where presented data is mean ± SD. Statistical analysis were calculated using one way ANOVA and ****p < 0.0001 (*n* = 3).

To further determine the effect of azithromycin against planktonic cells and biofilm formation on a solid surface, we performed a time-kill kinetic study using a catheter piece as a solid surface objective, as described earlier (Kumar et al., 2019), although azithromycin showed a substantial 1.86 log_10_ reduction in planktonic cell counts at 6 h as compared to the drug-free control and but no effect at 24 h post-incubation (Figure 2A and Supplementary Figure 3). Possibly, over-expression of multidrug efflux pumps could be the reason of its poor activity against *P. aeruginosa* in LB media, as potent-activity of azithromycin has been observed in the presence of known efflux pump inhibitors or eukaryotic media (RPMI 1640) (Buyck et al., 2012). Despite the high bacterial cell counts in LB media (Figure 2A), azithromycin exhibited a strong activity against *P. aeruginosa* PA-14 biofilm (Figure 2B). A simple macroscopic and quantitative analysis 24 h after incubation revealed that *P. aeruginosa* PA-14 formed a strong solid-surface biofilm on catheter pieces (Figures 2C,D), whereas in the presence of azithromycin, no solid-surface biofilm formed, suggesting that, despite having a limited activity against the planktonic cells of *P. aeruginosa* in LB media, azithromycin efficiently impeded the planktonic cells from leading to biofilm formation, possibly by inhibiting the synthesis of the extracellular biofilm matrix required for solid-surface biofilm formation. To confirm our hypothesis, we used SEM to visualize biofilm formation at the cellular level in a drug-free control and the presence of azithromycin. As shown in Figure 2C, *P. aeruginosa* cells were embedded in an extracellular matrix to form a solid-surface biofilm in a drug-free control, whereas no extracellular matrix was observed in the presence of azithromycin (Figure 2D). Hence, biofilm formation was significantly inhibited. These lines of evidence support the hypothesis that azithromycin inhibits biofilm formation by impairing the ability of *P. aeruginosa* to produce its biofilm matrix.

Development of the solid-surface-associated biofilm is controlled by *pel* genes in *P. aeruginosa*, as a *pel* gene mutant was found defective in synthesizing an extracellular biofilm matrix and biofilm formation (Sakuragi and Kolter, 2007), and the expression of *pel* genes is regulated by 3-O-C_1__2_ homoserine lactone (autoinducer of the *las* QS signaling system, synthesized by *lasI*) (Sakuragi and Kolter, 2007).

**FIGURE 3:**
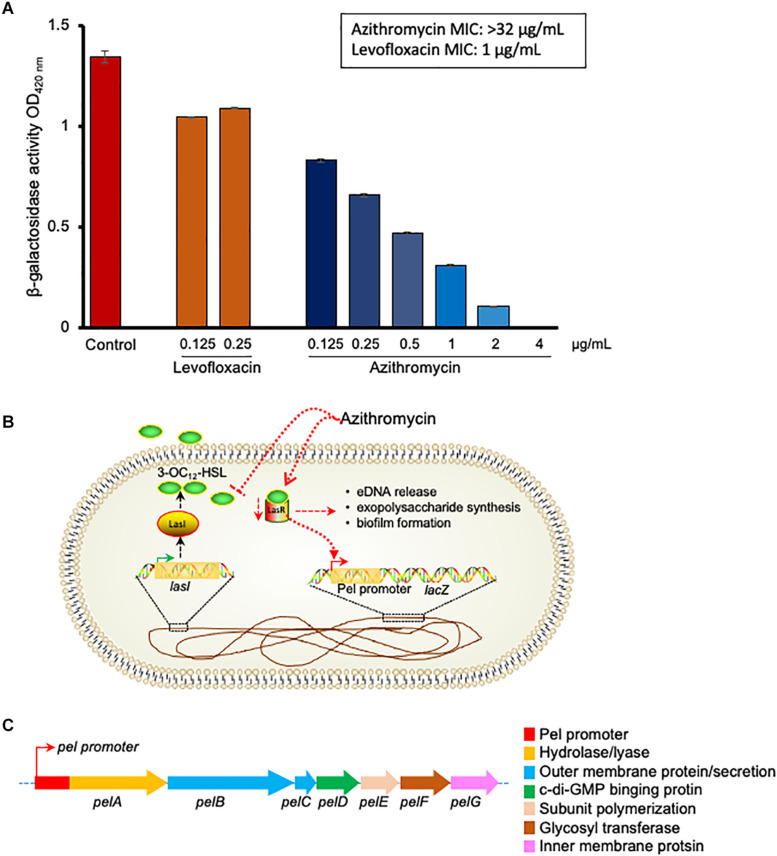
p-galactosidase activity of *P. aeruginosa*G2 recombinant construct containing pel promoter-lacZ transcriptional fusion in presence of different concentration of azithromycin, levofloxacin or no-drug control **(A)**. The MIC of azithromycin and levofloxacin was > 32 and 1 ng/mL, respectively, against *P. aeruginosa*G2. Proposed mechanism of biofilm and *pel* genes inhibition by azithromycin **(B)**.Genetic structure of *pel* genes in *P. aeruginosa* **(C)**.

To understand the biofilm inhibition mechanism of azithromycin, we evaluated the β-galactosidase activity of a recombinant mutant containing a promoter less *lacZ* gene under promoter control of *pel* gene (Sakuragi and Kolter, 2007), in the absence and presence of different azithromycin concentrations. Azithromycin showed a dose-dependent inhibition of β-galactosidase activity with IC_50_ 0.170 μg/ml (Figure 3). In addition, to confirm the selectively of azithromycin from the Las QS system, we also evaluated the sub-MIC concentrations of levofloxacin. As expected, levofloxacin did not influence the β-galactosidase activity of mutant (Figure 3A). Taken together, these results indicate that azithromycin inhibits *pel* gene transcription by inhibiting the *las* QR signaling system (Figures 3B,C), resulting in the inhibition of *P. aeruginosa* biofilm formation.

### *In vivo* Efficacy Against *P. aeruginosa* in a Rat Biofilm Model

**FIGURE 4:**
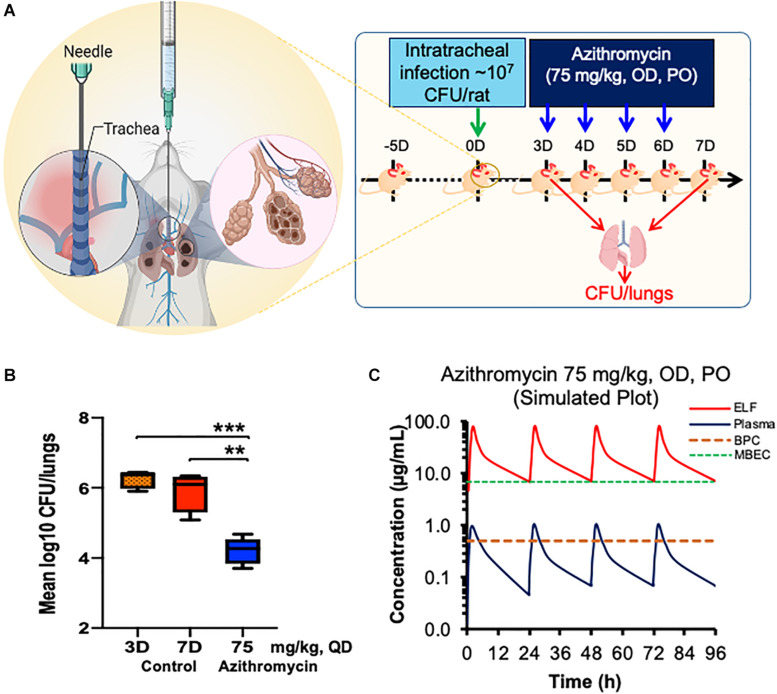
Efficacy of azithromycin in chronic rat pulmonary infection model of P. *aeruginosa* PA-14.Chronic pulmonary was caused using *P aeruginosa*PA-14 in rats via the intratracheally route and treatment was started 3 days post infection with azithromycin by PO route **(A)**. Bacterial lungs loads were determined post 22 h of last dose and compared with those of placebo treated **(B)**. Stimulated plasma and ELF concentration of azithromycin at 75 mg/kg, PO for 4 days was calculated using WinNonlin **(C)**. The asterisks indicates a significant difference compared with those of control group (**P < 0.01, ***P < 0.001). CFU, Colony Forming Unit; OD, Once a Day; PO, Per Orem (Oral administration); ELF, Epithelial Lining Fluid; BPC, Biofilm Preventive Concentration; MBEC, Minimal Biofilm Eradication Concentration.

The experimental plan used for testing the efficacy of azithromycin at a human-equivalent dose is shown in Figure 4. The treatment was implemented 3 days post-infection and given once daily for a period of 4 days (Figure 4A). All the infected animals in the control group exhibited consistent bacterial counts. At 4 days, the placebo treatment resulted in no significant change in bacterial load, whereas treatment with azithromycin at 75 mg/kg, q24h resulted in a significant 1.67 log_10_ reduction in bacterial counts as compared to the placebo control (Figure 4B).

### Pharmacokinetics of Azithromycin in *P. aeruginosa*-Infected Rats

The plasma and lung tissue kinetics of azithromycin in the SD rats infected with *P. aeruginosa* PA-14 are presented in Figure 4C and Table 1. The plasma and lung tissues were harvested from infected animals at different time-points of up to 24 h post-dosing, and the stimulated plasma and lung tissue (EFL) profile was prepared to understand the pharmacokinetics of azithromycin in infected animals. Although *C*_max_ in plasma and lung tissues was observed 2 h after administration, azithromycin showed comparatively higher concentrations in lung tissues. The lung ELF/plasma ratios of *C*_max_ and AUC_inf_ were 81.05 and 85.96, respectively. The graph shows that the simulated lung concentrations of azithromycin at 75 mg/kg, q24h dose were substantially higher than the BPC and MBEC values of *P. aeruginosa* biofilms throughout the treatment (Figure 4C).

**TABLE 1:** Pharmacokinetic parameters of azithromycin at 75 mg/kg, PO dose in Wister rats infected with *P. aeruginosa* PA-14.

Parameters	Azithromycin Plasma	Lung ELF
c_max_(μg/L)	0.95 ± 0.30	82.00 ± 26.10
Tmax (h)	2.00 ± 0.00	2.00 ± 0.00
AUC_0_._24_ (μg.h/L)	5.91 ± 0.62	508.11 ± 53.54
AUC_inf_ (μg.h/L)	6.92 ± 0.91	594.88 ± 77.68

**Values are presented as mean ± SD, n = 3.**

## Discussion

Biofilm formation within the lungs of CF patients is the hallmark of *P. aeruginosa* pathogenesis (Singh et al., 2000), as it substantially reduced the bacterial susceptibility to antimicrobial agents and the host immune system compared to planktonic cells (Smith and Iglewski, 2003; Mulcahy et al., 2014). Biofilm infections (including CF infections) tend to be chronic and difficult to eradicate and remain a matter of great concern, which has generated the need for alternative treatment strategies. The most striking findings with azithromycin in the present investigation were its capacity to inhibit *P. aeruginosa* biofilm formation and reduce the solid-surface biofilm attachment almost to a lower detection limit. Interestingly, the SEM of solid-surface biofilm in the presence of azithromycin shows a substantially reduced extracellular biofilm matrix and attachment to a solid surface to form a biofilm, which is in accordance with the findings of other studies (Mizukane et al., 1994; Ichimiya et al., 1996). Although azithromycin is well known to show a potent activity against *P. aeruginosa* biofilm, its evaluation in chronic respiratory *P. aeruginosa* biofilm infection model has been relatively understudied. There is a pressing need to determine its bactericidal activity on biofilms to facilitate its clinical use.

To evaluate the potential mechanism of azithromycin to inhibit biofilm formation, we developed a refined and chronic lung rat model of *P. aeruginosa* to mimic the clinical and chronic *P. aeruginosa* infection conditions in patients (Hraiech et al., 2012; Chawanpaiboon et al., 2019). The most important features of the rat chronic *P. aeruginosa* infection model were consistent and reproducible bacterial lung counts in infected animals with no acute morbidity but only delayed mortality of infected rats within 7–14 days if left untreated (data not shown), which makes it ideal to evaluate efficacy against chronic disease. These findings align with those of CF patients who suffer from chronic *P. aeruginosa* infections and show high mortalities. In this study, we evaluated the efficacy of azithromycin at 75 mg/kg, once daily, which was equivalent to 500 mg every 24 h in humans (Beringer et al., 2005; Cipolli et al., 2012; Dalhoff, 2014). To mimic the clinical chronic infection conditions, the treatment was initiated 3 days post-infection with 75 mg/kg, q24h of azithromycin for 4 days by the PO route. This treatment regimen resulted in a significant reduction in the lungs’ bacterial counts compared to the placebo-treated control.

To understand the pharmacodynamics of potent *in vivo* activity of azithromycin, we performed pharmacokinetics of azithromycin in the SD rats infected with *P. aeruginosa*. Interestingly, we observed a significantly higher concentration of azithromycin in the lungs of the infected animals, which was found to be well above the BPC and MBEC values of azithromycin. This explains the potent *in vivo* efficacy of azithromycin in treating the chronic lung infection model of *P. aeruginosa*. Our data are in accordance with the clinical findings that azithromycin treatment improves lung function in CF patients chronically infected with *P. aeruginosa* either by inhibiting biofilm formation (Saiman et al., 2003; Hansen et al., 2005) or suppressing the growth of susceptible bacteria.

**FIGURE 5:**
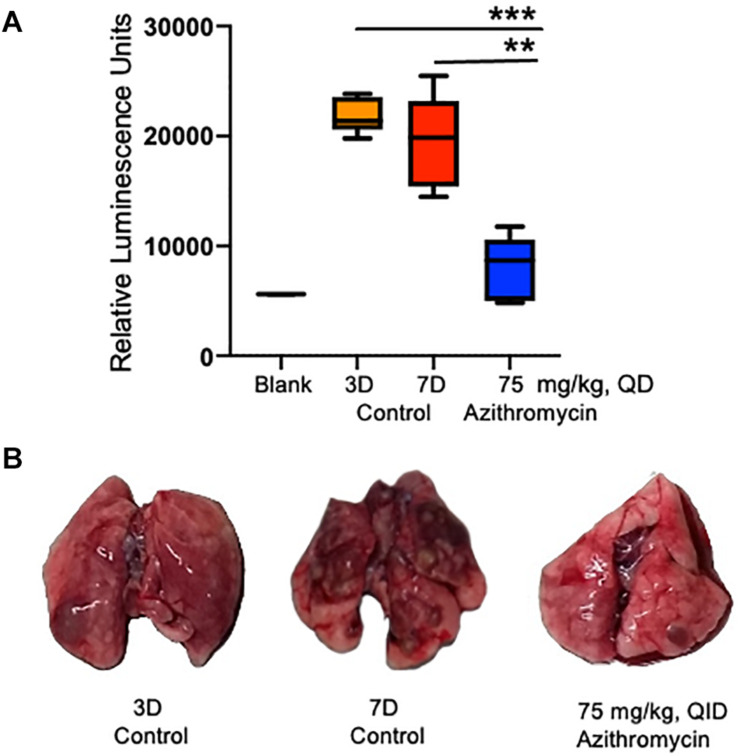
Detection of QS molecule in the epithelial lining fluid (ELF) of infected rats. QS molecule was determined 3 days post-infection, when the treatment was started and at 7 days post-infection in the placebo control and azithromycin treated rats **(A)**. Representative lungs photos of 3D, 7D and azithromycin treated rats **(B)**. The asterisks indicates a significant difference compared with those of 7D control group (**P < 0.01, ***P < 0.001).

The bacterial biofilm matrix is generally composed of exopolysaccharides, proteins (Sutherland, 2001), and e-DNA (Cherny and Sauer, 2019), which is required for both structural integrity and protective barrier to the harsh environment (Mulcahy et al., 2014). Three main exopolysaccharides (*psl*, *pel*, and alginate) have been identified in *P. aeruginosa* (Sutherland, 2001). Among these, *pel* gene cluster regulated by LasI/LasR (3-O-C_12_ HSL molecule synthesized by *lasI*) was found important for bacterial attachment to solid surfaces and biofilm formation (Sakuragi and Kolter, 2007). A dose-dependent inhibition of the *pel* promoter by azithromycin in our study suggests that azithromycin affects biofilm formation by inhibiting the synthesis of the QS molecule (3-O-C_12_). In addition to the regulation of *in vitro* biofilm, the QS molecules are also critical for the infectivity of *P. aeruginosa*, as QS mutant showed impaired chronic infectivity compared to wild-type *P. aeruginosa* (Willcox et al., 2008). Interestingly, a comparatively reduced level of QS molecules (a marker of biofilm formation) in the ELF of animals treated with azithromycin further suggests the important role of the QS system during chronic *P. aeruginosa* infection (Figure 5) and indicates that azithromycin modulates the biofilm by inhibiting the synthesis of 3-O-C12 HSL, a known marker of biofilm formation (Barr et al., 2015). Our results with rats with chronic *P. aeruginosa* infection demonstrated that azithromycin significantly improved the pulmonary *P. aeruginosa* clearance, possibly by interfering with QS systems and crippling of the exopolysaccharides of the biofilm synthesized by the *pel* proteins. Besides inhibiting the virulence factors, azithromycin is known to have multiple effects against *P. aeruginosa*: (a) enhanced activity in the presence of serum, which could contribute to bacterial killing during chronic pulmonary infection (Lucchi et al., 2008), (b) anti-inflammatory activity that could help to reduce the inflammation at infection sites (Zeng et al., 2016; Zimmermann et al., 2018), (c) biofilm inhibitory or eradicative activity by modulating the synthesis of signaling molecules, such as the QS system, ci-di-GMP, and AHL (Favre-Bonte et al., 2003; Nalca et al., 2006; Barr et al., 2015; Ghosh et al., 2020), and (d) anti-virulence activity that could impair *P. aeruginosa* cells to produce extracellular biofilm matrix. These multiple effects of azithromycin could together be important mediators for its potent *in vivo* efficacy, or is there something else that drives the efficacy against *P. aeruginosa* in chronic CF patients? Could it be that host immune modulation leads to killing of bacterial cells or inhibition/eradication of biofilm and exposing the bacterial cells to an accumulated azithromycin concentration in the lung? These could be interesting avenues for investigation for future research.

In the present study, we reported the detailed biofilm preventive effect of azithromycin. However, our study has several limitations. First, the *in vitro* biofilm assays revealed that azithromycin shows better BPC than the MPEC, which was also supported by the reduced production of extracellular biofilm matrix in the presence of azithromycin, while the *in vivo* efficacy of azithromycin was evaluated against chronic *P. aeruginosa* infection (preformed biofilms). Second, the *in vivo* study was performed only at a single human-equivalent dose. Therefore, further detailed studies are required to access the preventive and eradicative biofilm effect of azithromycin at multiple doses to understand its clinical utilities in CF patients.

In summary, we demonstrated that azithromycin repressed the expression of 3-O-C_12_ (QS system) and inhibits biofilm matrix production by influencing the expression of *pel* genes. Furthermore, azithromycin treatment of rats with chronic *P. aeruginosa* infection significantly improved the bacterial clearance from chronic infection compared to placebo controls. Taken together, the repression of bacterial virulence, the bactericidal effect on biofilm formation, and the enhanced serum sensitivity after a prolonged exposure to azithromycin may be responsible for the potent efficacy observed in our rats with chronic infection and in patients with CF.

## Data Availability Statement

The original contributions presented in the study are included in the article/Supplementary Material, further inquiries can be directed to the corresponding author/s.

## Ethics Statement

The animal study was reviewed and approved by the Institutional Animal Ethics Committee at Daiichi Sankyo Pharma Pvt. Ltd., Gurgaon.

## Author Contributions

MK and MR conceived the study, performed statistical analyses, and generated graphs. MK, MR, TM, TKB, VJ, SS, and MP performed the *in vitro* and *in vivo* studies. TC performed the PK studies. MP, DJU, and NM did the project management. MK, MR, and SA wrote the first draft of manuscript. All authors contributed to manuscript editing, discussed the results, and approved the final submitted version.

## Conflict of Interest

MK, MR, TM, TKB, VJ, TC, SS, MP, DJU, and NM were employed by the company Daiichi Sankyo Pharma Pvt. Ltd., Gurgaon. The remaining author declares that the research was conducted in the absence of any commercial or financial relationships that could be construed as a potential conflict of interest.
