# Supplementary figures and images for: Azithromycin Exhibits Activity Against Pseudomonas aeruginosa in Chronic Rat Lung Infection Model
Source: Front Microbiol. 2021 Apr 23;12:603151. doi: 10.3389/fmicb.2021.603151 (PMC8102702; doi:10.3389/fmicb.2021.603151)

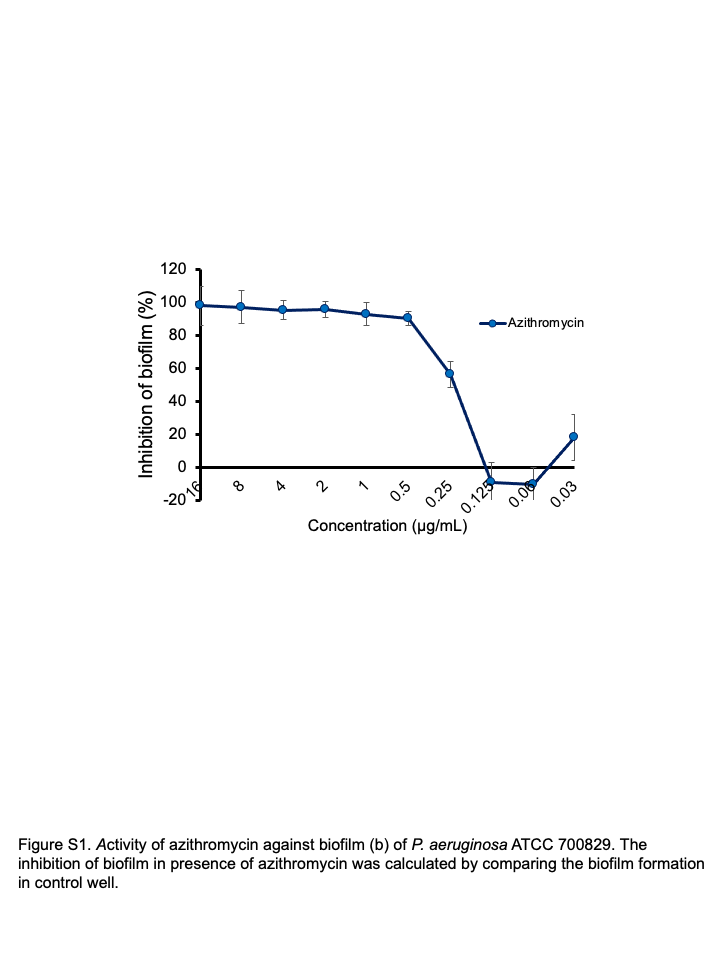

Supplement: Supplementary file 1 [file Image_1.tiff]

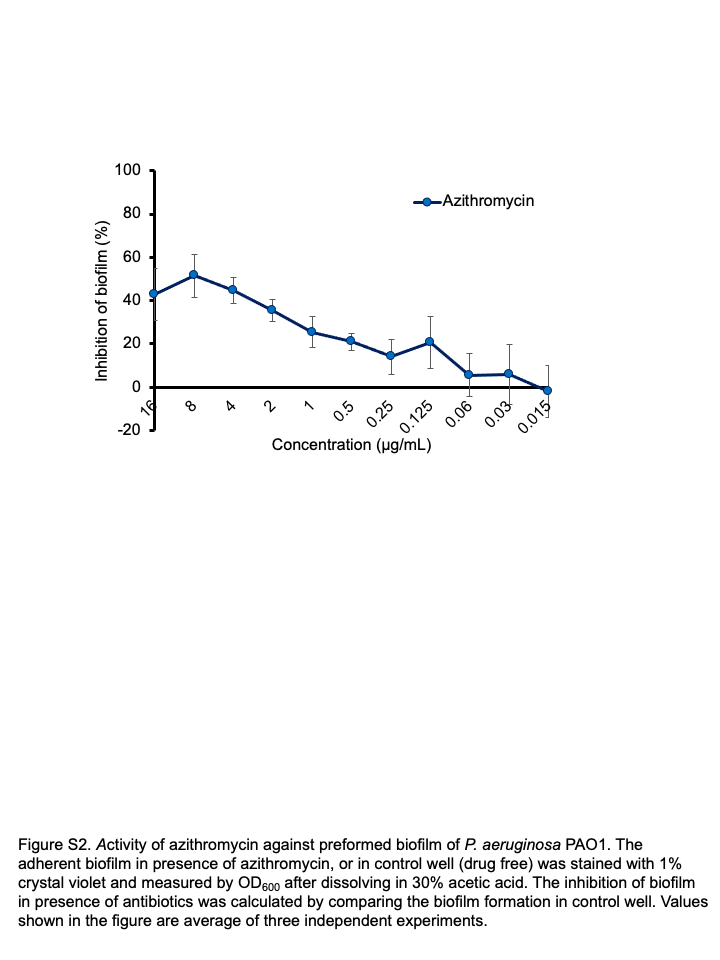

Supplement: Supplementary file 2 [file Image_2.tiff]

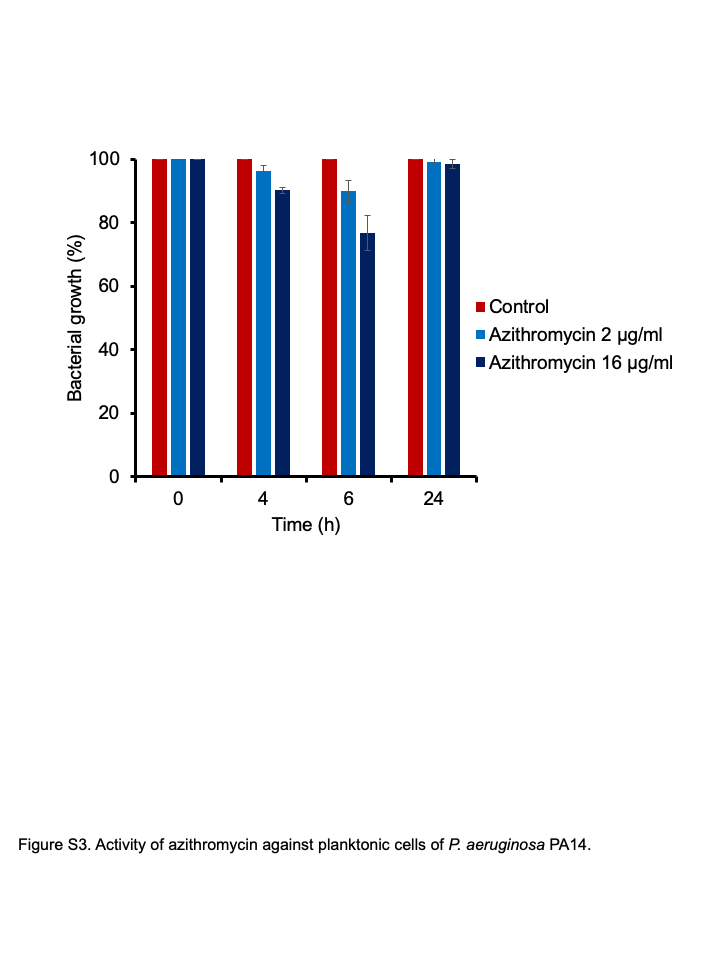

Supplement: Supplementary file 3 [file Image_3.tiff]
